# Optimal Energy Delivery, Rather than the Implementation of a Feeding Protocol, May Benefit Clinical Outcomes in Critically Ill Patients

**DOI:** 10.3390/nu9050527

**Published:** 2017-05-21

**Authors:** Chen-Yu Wang, Chun-Te Huang, Chao-Hsiu Chen, Mei-Fen Chen, Shiu-Lan Ching, Yi-Chia Huang

**Affiliations:** 1 Department of Critical Care Medicine, Taichung Veterans General Hospital, Taichung 40705, Taiwan; chestmen@gmail.com (C.-Y.W.); Huangchunte@gmail.com (C.-T.H.); slcvgh@gmail.com (S.-L.C.); 2 Department of Nursing, Hung Kuang University, Taichung 43302, Taiwan; 3 Department of Food and Nutrition, Taichung Veterans General Hospital, Taichung 40705, Taiwan; hsiu@vghtc.gov.tw; 4 Department of Nursing, Taichung Veterans General Hospital, Taichung 40705, Taiwan; zox1120@yahoo.com.tw; 5 Department of Nutrition, Chung Shan Medical University, Taichung 40201, Taiwan; 6 Department of Nutrition, Chung Shan Medical University Hospital, Taichung 40201, Taiwan

**Keywords:** energy delivery, feeding protocol, clinical outcomes, critically ill patients

## Abstract

Malnutrition is common in intensive care units (ICU), and volume based feeding protocols have been proposed to increase nutrient delivery. However, the volume based approach compared to trophic feeding has not been proven entirely successful in critically ill patients. Our study aimed to compare the clinical outcomes both before and after the implementation of the feeding protocol, and to also evaluate the effects of total energy delivery on outcomes in these patients. We retrospectively collected all patient data, one year before and after the implementation of the volume-based feeding protocol, in the ICU at Taichung Veterans General Hospital. Daily actual energy intake from enteral nutritional support was recorded from the day of ICU admission until either the 7th day of ICU stay, or the day of discharge from the ICU. The energy achievement rate (%) was calculated as: (actual energy intake/estimated energy requirement) × 100%. Two-hundred fourteen patients were enrolled before the implementation of the volume-based feeding protocol (pre-FP group), while 198 patients were enrolled after the implementation of the volume-based feeding protocol (FP group). Although patients in the FP group had significantly higher actual energy intakes and achievement rates when compared with the patients in the pre-FP group, there was no significant difference in mortality rate between the two groups. Comparing survivors and non-survivors from both groups, an energy achievement rate of less than 65% was associated with an increased mortality rate after adjusting for potential confounders (odds ratio, 1.6, 95% confidence interval, 1.01–2.47). The implementation of the feeding protocol could improve energy intake for critically ill patients, however it had no beneficial effects on reducing the ICU mortality rate. Receiving at least 65% of their energy requirements is the main key point for improving clinical outcomes in patients.

## 1. Introduction

Malnutrition is common in intensive care units (ICU); where the prevalence is approximately 40 to 80% [1,2,3]. The stress experienced during critical illness, along with insufficient intake of nutritional supplements trigger various mechanisms which enhance prolonged catabolism [4]. Therefore, adequate nutritional supports are extremely important and required for critically ill patients to help them meet their metabolic needs. Enteral feeding is an important nutritional support method for critically ill patients [5,6], and adequate enteral feeding has been shown to reduce both mortality and nosocomial infection [7,8,9]. If patients receive inadequate feeding, it may result in poor clinical outcomes, including an increased infection rate, a prolonged length of ICU and hospital stay, and a delayed weaning from mechanical ventilation [10,11,12,13,14]. Although in theory, providing critically ill patients with at least 80% of their predicted calories and more than 1.2 g/kg/day of protein could improve their clinical outcomes [15,16,17], critically ill patients generally receive inadequate feeding [18,19,20]. To improve the provision of energy and protein in critically ill patients, the volume based feeding protocol has been implemented to enhance the infusion rate, in order to cover the interruption in delivery. This strategy has been proven to be safe, and could therefore meet energy requirements for critically ill patients [21,22]. Recently introduced provision guidelines for critically ill adult patients, as set by the Society of Critical Care Medicine (SCCM) and the American Society for Parenteral Enteral Nutrition (ASPEN) [6], also highlight the importance of the volume-based feeding protocol. Additionally, they recommend that this feeding protocol be used in the adult ICU, in order to reach the goal of calories provided [6]. Based on the beneficial effects that volume-based feeding offers, the feeding protocol has been implemented in our ICU since June 2015 at Taichung Veterans General Hospital (TVGH) in Taichung, Taiwan.

On the other hand, not all studies proved that there were beneficial effects of volume-based feeding protocol for critically ill patients. Some studies indicated that volume-based feeding did not improve clinical outcomes, when compared with either trophic feeding or permissive underfeeding in critically ill patients [23,24]. Even though the subject inclusion criteria (i.e., age, body mass index (BMI, kg/m^2^), and the length of ICU stay) may not exactly be compatible among studies, these studies have shown that even minimal amounts of nutritional support (trophic nutrition) may have beneficial effects [25,26]. Due to the fact that conflicting results still exist regarding the optimal feeding amounts for critically ill patients, it would be worthwhile to assess the clinical outcomes both before and after the implementation of the volume-based feeding protocol. Therefore, the purpose of this study was to compare the clinical outcomes before and after feeding protocol implementation. In addition, the effect of the amount of energy delivery on clinical outcomes was also evaluated.

## 2. Materials and Methods

### 2.1. Study Design

This was a retrospective cross-sectional study, conducted in the ICUs of TVGH, which is a tertiary medical center located in central Taiwan. 

### 2.2. Feeding Protocol

**Figure 1:**
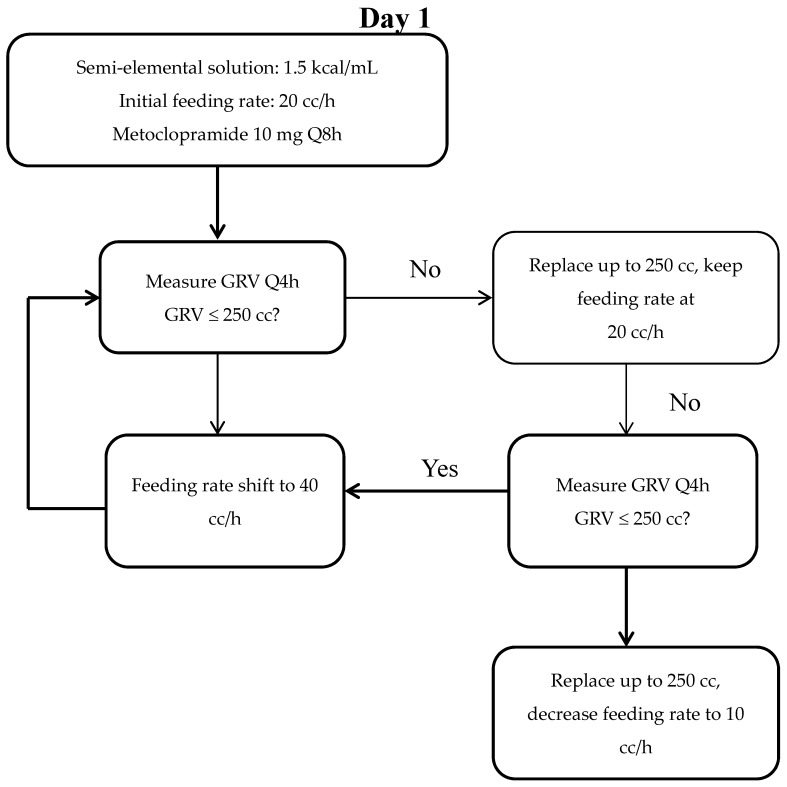

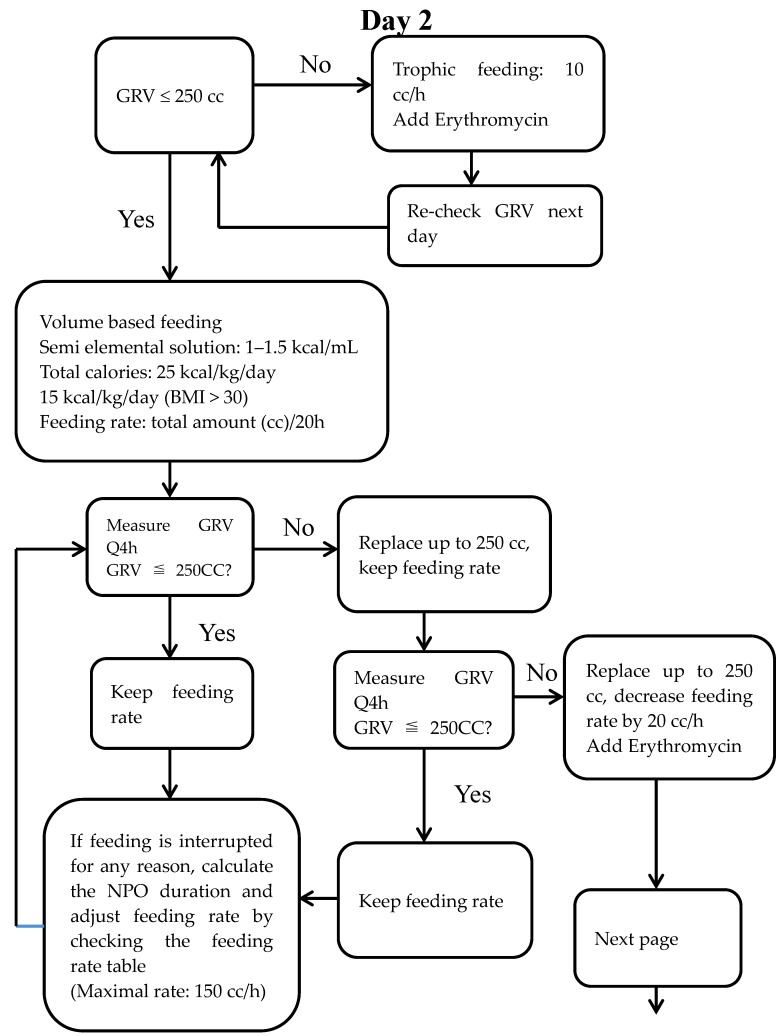

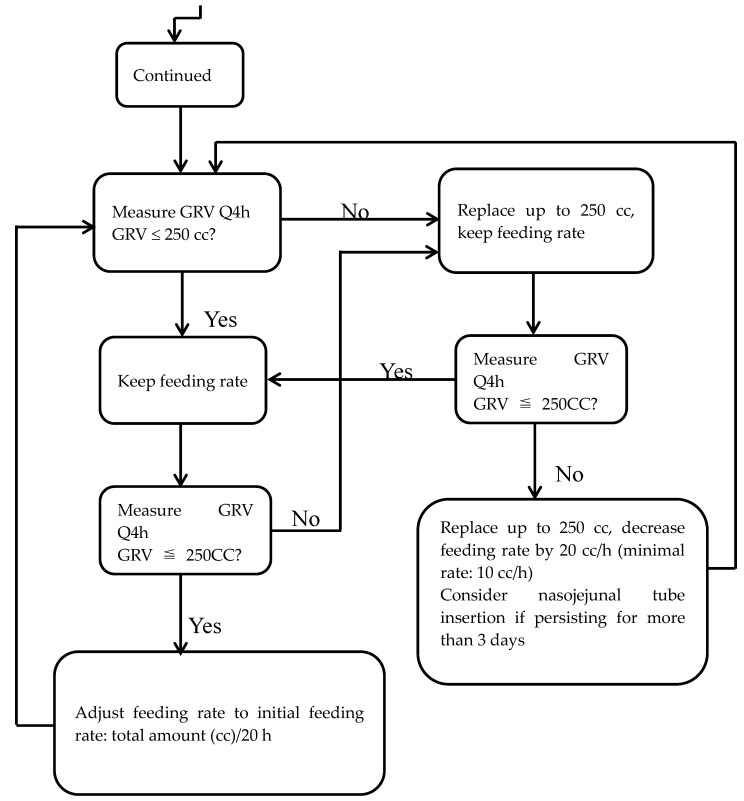
Volume-based feeding protocol flow chart. GRV, gastric residual volume, NPO, nil per os.

The volume-based feeding protocol (Figure 1) has been implemented in TVGH’s ICU since June 2015. This protocol has been slightly modified from the PEP uP protocol [27], and consists of volume-based feeding with a target rate, with the start to feeding at day 1, and with an advanced to target feeding rate on day 2. The enteral nutrition formula began with a semi-elemental formula, prophylactically used metoclopramide given intravenously at 10 mg per 8 h. Erythromycin at 250 mg per 12 h was added if gastric residual volumes (GRVs) persistently exceeded 250 mL within two episodes. The threshold of GRVs was set at 250 mL, while each patient’s protein need was provided at a minimum of 1.2 g/kg/day.

### 2.3. Patients

We retrospectively collected each patient’s data one year prior to (June 2014 to May 2015) and after (June 2015 to April 2016) the implementation of the volume-based feeding protocol. The inclusion criteria were an age older than 20 years, receiving only enteral nutritional support, and receiving at least 48 h of mechanical ventilation. Patients were excluded when they experienced gastroenteral bleeding, were being fed with percutaneous endoscopic gastrostomy or jejunostomy, or were receiving nil per os through a physicians’ order within the first seven days of admission to the ICU. Patients were then divided into a pre-feeding protocol group (pre-FP group) and feeding protocol group (FP group). In a post hoc analysis, we pooled all patients and divided them into survivor and non-survivor groups based on their mortality outcomes. This study was approved by the Institutional Review Board of TVGH (IRB No. CE17074B). It was agreed that informed consent be waived by the Institutional Review Board of TVGH, since the data was retrospectively collected and de-linked.

### 2.4. Data Collection and Energy Intakes

Data on patients’ age, gender, BMI, clinical outcomes including the severity of illness (acute physiology and chronic health evaluation II, APACHE II score), length of ventilator dependence, length of hospital and ICU stays, mortality, and comorbidities, were carefully reviewed and collected from the medical charts. Mortality was determined as the primary end point. 

Daily actual energy intake from enteral nutritional support was recorded from the day of ICU admission until the 7th day of the ICU stay, or the day of discharge from the ICU. The amount of energy intake represented in the result is the mean amount of energy delivered in each group. The daily estimated energy requirement was simply estimated through a weight-based equation (25 kcal/kg/day). The energy achievement rate (%) was calculated as: (actual energy intake/estimated energy requirement) × 100%.

### 2.5. Statistical Analysis

Our group size was based on the requirements for the detection of a difference of 10% in the mortality rate between pre-FP and FP groups with a power of 80%, and a 2-sided test with an α of 0.05. The required sample size was a minimum of 313 subjects. All data were analyzed by the SAS statistical software package (version 9.3; Statistical Analysis System Institute Inc., Cary, NC, USA). Continuous variables were described using the mean and standard deviation and the Student’s *t*-test or Mann-Whitney Ran Sum test, in order to compare the groups for significance. Categorical variables were described with frequency and percentage rates, using Chi-square test or Fisher’s exact test to examine the significance. Multivariate logistic regression was used to estimate the odds ratios and 95% confidence intervals for mortality. All tests were performed with two sided tests, where *p*-value < 0.05 was considered as statistical significance.

## 3. Results

A total of 412 patients (169 women, 243 men) were included in this study, where 214 patients (89 women, 125 men) were enrolled before the implementation of the volume-based feeding protocol (pre-FP group), while 198 patients (80 women, 118 men) were enrolled after the implementation of the volume-based feeding protocol (FP group). Patients’ demographics, clinical outcomes, and energy intakes in the pre-FP and FP groups are shown in Table 1. Patients’ most common comorbidities were diabetes mellitus, liver cirrhosis, uremia, central nervous system, immunocompromised disorders, and chronic lung disease. Except for age difference, there were no significant differences in gender, BMI, clinical outcomes, or mortality rate between pre-FP and FP groups. Not surprisingly, patients in the FP group had significantly higher actual energy intakes and achievement rates when compared with patients in the pre-FP group. However, fewer patients in the FP group suffered from liver cirrhosis and chronic lung diseases than those in the pre-FP group. 

**Figure 2:**
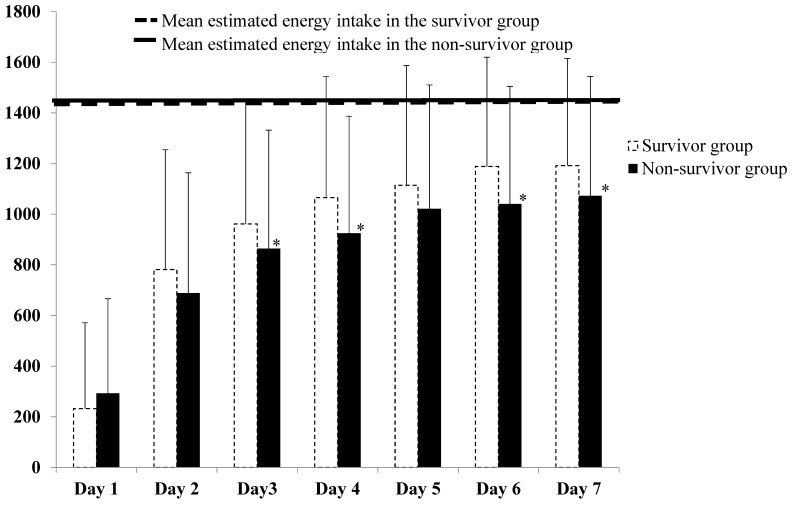
Mean estimated energy intake and actual energy intake in the survivor and non-survivor group from day 1 to day 7. * Values are significantly different between the survivor and non-survivor groups; *p* < 0.05.

There were no significant differences in age, gender, BMI, and APACHE II scores between the survivor and non-survivor patients (Table 2). However, the non-survivor patients displayed poorer clinical outcomes, including longer lengths of ventilator dependency, ICU, and hospital stays when compared with the survivor patients (Table 2). In addition, the survivor patients had a significantly higher actual energy intake and energy achievement rate than the non-survivor patients (Table 2). The survivor patients began consuming significantly higher energy at days 3 through 7 in the ICU, when compared to the non-survivor patients (Figure 2).

To evaluate the association between mortality and energy intake, we calculated the mortality risk through the percentage of the energy achievement rate according to the distribution of all the patients (Table 3). There was a significant association between the energy achievement rate and mortality, with or without adjusting for the potential confounders. We further observed that when the energy achievement rate was less than 65%, it would significantly increase the mortality rate with or without adjusting for any potential confounders. 

## 4. Discussion

Heyland et al. [21] introduced the volume-based feeding protocol for critically ill patients, and later demonstrated that this protocol could enhance the delivery of both the energy and protein intake by reducing feeding interruptions while patients were in the ICU [27]. Since June 2015, our medical team has started to implement a similar feeding protocol in our medical ICU. Although the achievement rate of energy delivery did significantly improve from 57.7% to 70.3% after the protocol implementation, our patients’ clinical outcomes did not significantly improve. This finding truly came as a bit of a disappointment. However, our results should not be used as a claim that the volume-based feeding protocol is not relevant when attempting to achieve improved clinical outcomes in the ICU, for the sole reason that the achievement of energy delivery was not fully attained in our critically ill patients. The SCCM and ASPEN [6] recently suggested that GRVs not be used as part of routine care to monitor ICU patients receiving enteral nutrition since GRVs did not correlate with adverse clinical outcomes. In our feeding protocol, the frequent measurement (checked with every 4 h) of GRVs might be time consuming and inducing unnecessary workload and anxiety among nurses, and could be a major obstacle to cause the energy delivery to not reach our targets. A large, multicenter and prospective trial (close to 8000 patients) showed that more than an 80% caloric intake was associated with a decrease in critically ill patients’ mortality rates [16]. Sufficient energy delivery is needed for critically ill patients in order to meet their metabolic needs; however, underfeeding still remains a major problem in the ICU [18,19,20]. Therefore, strategies have been developed to overcome the underfeeding problem. Previous studies have implemented evidence-based feeding algorithms which could achieve earlier feeding responses and increase greater nutritional adequacy, but they failed to demonstrate a significant reduction in ICU mortality rates [28,29]. However, it is important to note that energy delivery was not fully achieved to the target level during these studies and the present study. This might be a possible reason for the absence of an impact on the clinical outcomes even though we implemented a feeding protocol. The achievement of a target energy delivery seems to be very important for improving clinical outcomes. 

Martin et al. [30] indicated that an evidence-based algorithm for nutritional support (with a goal of at least 80% of the requirement within 72 h) could significantly reduce the length of hospital stays, while also slightly reducing mortality rates. Weijs et al. [31] has developed a nutrition algorithm to fully provide optimal protein and energy targets, which showed no significant reduction in ICU mortality rates, but did provide a 50% decrease in 28-day hospital mortality if both protein and energy targets could be simultaneously achieved. A large secondary analysis study by Elke et al. [32] indicated that when energy and protein delivery was closer to their recommended amounts through enteral nutrition, an association could be made showing a more favorable outcome. Heidegger et al. [33] and Petros et al. [34] indicated that energy delivery as close as possible to the 100% energy requirement could reduce the rate of nosocomial infections in critically ill patients. It seemed that energy delivery should be achieved at a level of at least 80% of the target goal, or the energy and protein targets will need to be simultaneously achieved in order to improve clinical outcomes. However not all studies, including the present one, support the belief that energy or protein targets must be fully achieved in order to improve clinical outcomes. It has been shown that even patients receiving 33–65% of their goal calories [35], initial trophic feeding (10 mL/h) for the initial 6 days of ventilation [24,36], trophic feeding (25% of energy goal) [37], or permissive underfeeding (40–60% of their energy requirement) [23] could be associated with a favorable clinical outcome when compared to those receiving full feeding. Based on these previous studies [23,35,36,37] and the present study, there will not be a significant difference in clinical outcomes between trophic feeding and full feeding, as long as a reasonable energy amount is delivered to critically ill patients. The challenge therefore is how to determine the reasonable energy delivery amount for critically ill patients.

Since the implementation of the feeding protocol may not have been the key strategy towards improving clinical outcomes in the present study, we pooled all the patients together to evaluate the optimal energy delivery amount needed in order to be associated with better clinical outcomes. Our results show that delivery of at least 65% of the energy requirement to critically ill patients could significantly reduce ICU mortality rates. However, the actual amount of energy delivery in the present study could prove to be even higher than 65% of the energy requirement, since we failed to calculate calories from glucose infusions, glucosaline, and Propofol due to the retrospective limitations. This amount of the energy requirement is higher than or equal to the previous trophic feeding results (25–65% of the energy requirement) [23,35,37], but lower than the amount (at least 80% of the energy requirement) recommended by other previous studies [16,17,33,34]. The recent ASPEN guidelines for adult critically ill patients suggest that any amount of parenteral nutrition energy should be discontinued, when the provision of enteral nutrition exceeds 60% of the target energy requirements [6]. Although there is still no agreement on the optimal amount of energy delivery for critically ill patients, we presently choose to suggest that at least 65% of the energy requirement should be delivered, in order to improve clinical outcomes of critically ill patients receiving enteral nutrition.

There were some limitations in this study. First, this was a single center study and was not prospectively designed. The data was retrospectively collected only one year before and after the implementation of the feeding protocol at different time periods. A beneficial effect of the feeding protocol may have been observed if the data had been collected over a longer period of time. Secondly, the target energy intake was not fully reached during the implementation of the feeding protocol, giving the possibility of causing the beneficial effect of the feeding protocol implementation to not be observed. Thirdly, our patients’ nutritional status could not be retrospectively assessed. A patient’s nutritional status may affect the amount of nutrient delivery, and previous studies have indicated that early enteral nutrition could provide a beneficial effect on the clinical outcomes in patients with a high nutritional risk [38,39]. Although the sample size was calculated and the final recruitment exceeded the required sample size, a larger sample size (several hundreds of patients) may offer more statistical power to deal with mortality and towards detecting the beneficial effects of the feeding protocol implementation. Nevertheless, the main strength of this study was in collecting a large sample size (412 medical ICU patients) from a single center. An additional strength is that we tried to examine the optimal energy delivery amount for critically ill patients for the purpose of reducing ICU mortality rates.

## 5. Conclusions

In conclusion, this retrospectively observational study showed that the implementation of the feeding protocol could improve energy intake for critically ill patients, but offered no beneficial effects on reducing ICU mortality rate. Other than the implementation of feeding protocols, we have to further improve our feed delivery, and patients receiving more than 65% of their energy requirement remain the key main point towards improving clinical outcomes. 

## Figures and Tables

**Table 1:** Patients’ demographic characteristics, clinical outcomes, and energy intakes before and after the implementation of a volume-based feeding protocol.

Variables	All (*n* = 412)	pre-FP Group (*n* = 214)	FP Group (*n* = 198)
Age (year)	69.4 ± 16.0	71.3 ± 15.0	67.4 ± 16.9 *
Gender (women/men)	169/2473	89/125	80/118
Weight (kg)	62.0 ± 14.6	61.6 ± 13.3	62.5 ± 5.9
Body mass index (kg/m^2^)	23.95 ± 5.19	23.94 ± 4.77	23.97 ± 5.62
Length of ventilatory dependency (day)	20.9 ± 14.7	22.0 ± 16.7	19.7 ± 12.0
Length of ICU stay (day)	13.7 ± 7.3	13.2 ± 7.4	14.2 ± 7.2
Length of hospital stay (day)	30.5 ± 18.1	30.3 ± 19.3	30.6 ± 16.6
APACHE II score	28.0 ± 5.9	27.8 ± 5.5	28.2 ± 6.2
Mortality (*n*, %)	117, 28.4%	55, 25.7%	62, 31.3%
*Energy Intakes*			
Estimated energy requirement (kcal/day)	1438.2 ± 223.8	1419.3 ± 161.1	1458.6 ± 275.0
Actual energy intake (kcal/day)	908.0 ± 333.5	811.7 ± 347.4	1012.1 ± 283.9 *
Achievement rate (%)	64.3 ± 25.0	57.7 ± 25.1	70.3 ± 20.6 *
*Comorbidities* (*n*, %)			
Diabetes mellitus	159, 38.6%	81, 37.9%	78, 39.4%
Liver cirrhosis	37, 9.0%	27, 12.6%	10, 5.1% *
Uremia	52, 12.6%	26, 12.1%	26, 13.1%
Central nervous system disorders	96, 23.3%	48, 22.4%	48, 24.2%
Chronic lung diseases	55, 13.3%	40, 18.7%	15, 7.6% *
Immunocompromised disorders	130, 31.6%	74, 34.6%	56, 28.3%

Values are mean ± standard deviation. FP, feeding protocol; APACHE II, Acute Physiology and Chronic Health Evaluation II; ICU, intensive care unit. Achievement rate (%) = (actual energy intake/estimated energy intake) × 100%. * Values are significantly different between pre-FP and FP groups; *p* < 0.05.

**Table 2:** Patients’ demographic characteristics, clinical outcomes, and energy intakes in survivor and non-survivor groups.

Variables	Survivor Group (*n* = 295)	Non-Survivor Group (*n* = 117)
Age (year)	68.7 ± 16.4	71.3 ± 15.1
Gender (Female/Male)	127/168	42/75
Weight (kg)	61.9 ± 14.5	62.4 ± 14.8
Body mass index (kg/m^2^)	23.90 ± 5.39	24.08 ± 4.68
Length of ventilatory dependency (day)	20.2 ± 14.7	22.7 ± 14.5 *
Length of ICU stay (day)	12.9 ± 6.5	15.7 ± 8.7 ^*^
Length of hospital stay (day)	31.4 ± 17.7	28.1 ± 18.7 ^*^
APACHEII	27.6 ± 5.7	28.9 ± 6.2
*Energy Intakes* Estimated energy intakes (kcal/day)	1437.5 ± 217.6	1439.9 ± 239.6
Actual energy intakes (kcal/day)	933.7 ± 329.6	843.3 ± 335.8 *
Achievement rate (%)	66.1 ± 24.7	59.9 ± 25.3 *
*Comorbidities* (*n*, %) Diabetes mellitus	117, 39.7%	42, 35.9%
Liver cirrhosis	19, 6.4%	18, 15.4% *
Uremia	35, 11.9%	17, 14.5%
CNS disorder	74, 25.1%	22, 18.8%
Chronic lung disease	33, 11.2%	22, 18.8% *
Immunocompromised disorders	81, 27.5%	49, 41.9% *

Values are mean ± standard deviation. APACHE II, Acute Physiology and Chronic Health Evaluation II; ICU, intensive care unit. Achievement rate (%) = (actual energy intake/estimated energy intake) × 100%. * Values are significantly different between the survivor and non-survivor groups; *p* < 0.05.

**Table 3:** Adjusted odds ratios of mortality.

	No Factors Adjusted for			Additional Factors Adjusted for Age, Gender, BMI			Additional Factors Adjusted for Age, Gender, BMI and APACHE II		
	OR	95% CI	*p*	OR	95% CI	*p*	OR	95% CI	*p*
Achievement rate (%)	0.4	(0.15–0.88)	0.03	0.4	(0.15–0.89)	0.03	0.4	(0.15–0.93)	0.04
Achievement rate (%)									
>65%	1		0.02	1		0.03	1		0.04
≤65%	1.7	(1.10–2.62)		1.7	(1.07–2.58)		1.6	(1.01–2.47)	

OR, odds ratio; BMI, body mass index.

## Abbreviations

The following abbreviations are used in this manuscript:

APACHE II	acute physiology and chronic health evaluation II
ASPEN	American Society for Parenteral Enteral Nutrition
BMI	body mass index
FP	feeding protocol
GRVs	gastric residual volumes
ICU	intensive care units
SCCM	Society of Critical Care Medicine
TVGH	Taichung Veterans General Hospital
